# Correction, Vol. 10, No. 5

**DOI:** 10.3201/eid1209.C11209

**Published:** 2006-09

**Keywords:** correction, erratum

In “Syndromic Surveillance in Public Health Practice, New York City," by Richard Heffernan et al., errors occurred. On page 861, in Table 2, the numbers of visits indicated in the headings for columns 3, 4, and 5 are incorrect. In the corrected table, column 3, % age 13–39 y, n = 946,478; column 4, % age 40–64 y, n = 604,707; and column 5, % age >65, n = 259,615. Additionally, a footnote has been added to the column 2 heading: *Total number includes 7,266 visits for which patients’ ages were unavailable.

The corrected table appears in the updated article at http://wwwnc.cdc.gov/eid/article/10/5/03-0646-t2.htm

We regret any confusion these errors may have caused.

